# Host specificity and virulence of *Flavobacterium psychrophilum*: a comparative study in ayu (*Plecoglossus altivelis*) and rainbow trout (*Oncorhynchus mykiss*) hosts

**DOI:** 10.1186/s13567-024-01326-6

**Published:** 2024-06-12

**Authors:** Erina Fujiwara-Nagata, Tatiana Rochat, Bo-Hyung Lee, Delphine Lallias, Dimitri Rigaudeau, Eric Duchaud

**Affiliations:** 1 Department of Fisheries, Kindai University, Nara, Japan; 2 Université Paris-Saclay, INRAE, UVSQ, VIM, Jouy-en-Josas, France; 3 Université Paris-Saclay, INRAE, GABI, Jouy-en-Josas, France; 4 Université Paris-Saclay, IERP, INRAE, Jouy-en-Josas, France

**Keywords:** Pathogenicity, fish, serotype, O-antigen, experimental infection model, MLST, host–pathogen interaction, BCWD, rainbow trout fry syndrome

## Abstract

*Flavobacterium psychrophilum*, the causative agent of bacterial cold-water disease, is a devastating, worldwide distributed, fish pathogen causing significant economic loss in inland fish farms. Previous epidemiological studies showed that prevalent clonal complexes (CC) differ in fish species affected with disease such as rainbow trout, coho salmon and ayu, indicating significant associations between particular *F. psychrophilum* genotypes and host species. Yet, whether the population structure is driven by the trade of fish and eggs or by host-specific pathogenicity is uncertain. Notably, all *F. psychrophilum* isolates retrieved from ayu belong to Type-3 O antigen (O-Ag) whereas only very few strains retrieved from other fish species possess this O-Ag, suggesting a role in outbreaks affecting ayu. Thus, we investigated the links between genotype and pathogenicity by conducting comparative bath infection challenges in two fish hosts, ayu and rainbow trout, for a collection of isolates representing different MLST genotypes and O-Ag. Highly virulent strains in one host species exhibited low to no virulence in the other. *F. psychrophilum* strains associated with ayu and possessing Type-3 O-Ag demonstrated significant variability in pathogenicity in ayu, ranging from avirulent to highly virulent. Strikingly, *F. psychrophilum* strains retrieved from rainbow trout and possessing the Type-3 O-Ag were virulent for rainbow trout but not for ayu, indicating that Type-3 O-Ag alone is not sufficient for pathogenicity in ayu, nor does it prevent pathogenicity in rainbow trout. This study revealed that the association between a particular CC and host species partly depends on the pathogen’s adaptation to specific host species.

**Supplementary Information:**

The online version contains supplementary material available at 10.1186/s13567-024-01326-6.

## Introduction

Bacterial cold-water disease (BCWD), also known as rainbow trout fry syndrome, is a devastating bacterial fish disease [1]. *Flavobacterium psychrophilum*, the causative agent, affects high commercial value salmonid species such as rainbow trout (*Oncorhynchus mykiss*) and Atlantic salmon (*Salmo salar*) [2]. In addition, *F. psychrophilum* has been occasionally isolated from other non-salmonid freshwater fish, such as carp, sturgeon, sea lamprey, and eel [1, 3]. Ayu (*Plecoglossus altivelis*), an Osmeriformes, also appears highly susceptible to BCWD [4, 5]. Ayu is an important fish species in Japan, famous for game fishing (Tomozuri for anglers) but also produced in fish farms for human consumption. *F. psychrophilum* has been frequently associated with diseased ayu in farms and rivers in Japan and is responsible for heavy mortality and severe economic losses [4]. *F. psychrophilum* isolation from cultured ayu was also reported in Korea [6].

Multi-locus sequence typing (MLST) has been extensively used for epidemiological and population structure studies of *F. psychrophilum* [7–15]. They revealed that *F. psychrophilum* genotypes are usually specific for a given fish species (e.g., coho salmon, Atlantic salmon, rainbow trout, ayu), supporting the hypothesis of the pathogen’s adaptation to particular hosts. For instance, the majority of isolates retrieved from severe rainbow trout outbreaks mainly belonged to the clonal complex CC-ST10 regardless of their geographic origin [11, 13–15]. Another study pointed out that strains isolated from coho salmon and rainbow trout in Japan displayed the same sequence types (ST) as those isolated from the same fish species outside Japan [9]. However, *F. psychrophilum* strains isolated from ayu, which is not a member of the Salmonidae but Osmeridae, belong to STs that are clearly distinct from those identified in salmonid species such as rainbow trout and coho salmon. The ayu-derived strains were distributed across two lineages: CC-ST52 and CC-ST48-56. Notably, CC-ST48-56 is linked to strains isolated from cyprinid fish species in Japan and Germany [8, 9].

*F. psychrophilum* isolates can also be classified based on their serotypes [5, 16–19]. Multiplex PCR revealed a clear association between host fish species and a given serotype. Especially, Type-3 O-Ag is strongly associated with ayu whereas Type-1 and Type-2 are present in isolates retrieved from various salmonids species but are prevalent in rainbow trout isolates [20–23]. Meanwhile, some rare rainbow trout-derived *F. psychrophilum* strains possess Type-3 O-Ag [20]. However, the relative importance of O-Ag for *F. psychrophilum* pathogenicity and host specificity is unknown.

To date, the host associations of *F. psychrophilum* genotypes and serotypes have mostly been inferred from epidemiological studies of natural outbreaks and in vivo experimental infection studies are scarce [23–26]. They provide some evidence that specific genotypes of *F. psychrophilum* might have host-specific pathogenicity, especially in rainbow trout, whereas some isolates could potentially affect multiple salmonid species.

The aim of this study was to investigate the determinants that drive the association between *F. psychrophilum* genotypes and host species by a comparative analysis of pathogenicity using experimental infections in two fish species—namely, rainbow trout and ayu. We assessed the virulence of strains with or without Type-3 O-Ag through bath infection challenges using a selection of strains retrieved from various fish species.

## Materials and methods

### Bacteria and culture conditions

*F. psychrophilum* was routinely grown aerobically at 18 °C in glucose-supplemented tryptone yeast extract salts agar (FLPA) or broth (FLPB) (4 g Bacto Tryptone (BD), 0.4 g Yeast extract (BD), 0.2 g CaCl_2_・2H_2_O, 0.2 g MgSO_4_・7H_2_O, 0.5 g glucose, 15 g Bacto Agar (BD), 1 L distilled water, pH 7.2) [27]. Bacterial cells were revived from −80 °C on FLPA, a single bacterial colony was inoculated in 5 mL of FLPB and incubated for 24 h as a pre-culture. For ayu infection experiments, bacterial cultures were performed by inoculating 20 mL FLPB with the preculture at an optical density at 600 nm (OD600) of 0.01, then were incubated at 120 rpm until the OD600 reached approximately 0.2–0.4. For rainbow trout infection experiments, bacterial cultures were performed in 20 mL of FLPB and incubated at 200 rpm until OD600 of 1 that typically corresponds to the end of log phase of growth. Bacterial concentration was determined by colony counting on FLPA using tenfold serial dilutions method after 4 days of incubation at 18 °C.

### DNA extraction

An overnight culture of *F. psychrophilum* in FLPB was used for gDNA extraction using the Wizard genomic DNA purification kit (Promega). One μL of the extracted gDNA was used for MLST and multiplex PCR serotyping.

### MLST

MLST was performed following the methods described by Nicolas et al. and optimized by Fujiwara-Nagata et al. [8, 9]. Seven housekeeping genes (*atpA*, *dnaK*, *fumC*, *gyrB*, *murG*, *trpB*, *tuf*) were amplified by PCR. ExoSAP-IT (Thermo Fisher Scientific) was used for amplified products cleanup. Subsequently, the sequences were determined using the ABI 3130xl Genetic Analyzer (Applied Biosystems). The forward and reverse sequences were aligned using MEGA11 [28]. The aligned sequences were queried against the *F. psychrophilum* database in PubMLST [29] to obtain allele types (ATs). Sequence types (STs) were determined based on the combination of the seven allele types. Isolates sharing at least 5 ATs were assigned to the same clonal complex (CC). This relaxed parameter (5 instead of 6 shared ATs) defines extended CCs composed of single and double locus variants as previously reported [9, 12].

### Multiplex PCR serotyping

Multiplex PCR serotyping was performed following the methods described by Rochat et al*.* [20] and updated in Avendaño-Herrera et al. [22]. The FP0711 primer set targeting a highly conserved gene in *F. psychrophilum* was used as a positive control and the mPCR serotypes were classified into Type-0 to Type-4. According to the conventional serotyping scheme of Lorenzen and Olesen [16], Type-0, Type-3 and Type-4 correspond to FpT, Type-1 corresponds to Fd, Type-2 corresponds to Th. Type-3 likely corresponds to the O2 serotype defined by Izumi and Wakabayashi and Mata et al. [17, 18] and to serotype 7 described by Mata et al*.* [18]. Briefly, the 5 primer sets were added to the PCR mixture at a final concentration of 0.3 μM, and multiplex PCR was performed using Taq DNA polymerase (Takara) and the following PCR conditions: initial denaturation at 95 °C for 5 min, denaturation at 95 °C for 30 s, annealing at 52 °C for 30 s, extension at 72 °C for 60 s, repeated for 30 cycles, and a final extension at 72 °C for 10 min. The PCR products were electrophoresed on a 3% agarose gel at 100 V for 30 min, stained with ethidium bromide, and the sizes of the amplified products were determined.

### Rainbow trout bath challenges

Rainbow trout infection challenges were performed by immersion as previously described [30] using the rainbow trout isogenic line A36 maintained by INRAE [31]. Briefly, fish were reared at 10 °C in a recirculating aquaculture system with UV-treated dechlorinated water, then transferred to the BSL2 zone in 15 L tanks with flow water (1 renewal per hour) in similar rearing conditions for infection experiments. Fish were fasted for 48 h prior to infection. Overall, 18 strains were tested through 2 trials (Table 1A). Strain FRGDSA 1882/11 was included in the 2 trials to serve as positive control (1 replicate for each trial). Trial (I) was performed using groups of 30 fish (average body weight of 2.1 g) in duplicates (60 fish per strain). Biological independent bacterial cultures were used at OD600 ~ 1.2 (equivalent to 2 × 10^9^ CFU/mL) and were diluted 2000-fold into 10 L of aquarium water. For Trial (II), groups of 20 fish (average body weight of 2.5 g) in duplicates were infected using bacterial cultures at OD600 ~ 1 (equivalent to 1 × 10^9^ CFU/mL) and 1000-fold dilution into 15 L of aquarium water. Bacteria were maintained in contact with fish for 24 h. Throughout the experiments, water was maintained at 10 °C under continuous aeration and physical parameters (temperature, NH_4_^+^, pH, O_2_) were monitored immediately after the beginning and at the end of bacterial exposure, before refreshing the water. Sterile FLPB was used for the mock-infected control groups. After bath infection, fish were maintained in flow-through water at 10 °C and mortality was recorded twice a day for 25 days. *F. psychrophilum* concentration at 24 h post-exposure was determined by serial dilutions and plating of water samples on FLPA and CFU counting (Table 1A). This timepoint typically aligns with the highest bacterial concentration that fish are exposed to during the bath challenge, as indicated by prior research. Dead fish from each group were examined for the presence of *F. psychrophilum* in the spleen by plating tissue homogenates on FLPA and visually inspecting the appearance of bacterial yellow colonies.

**Table 1:** ***F. psychrophilum* experimental infection schemes**

Species origin^a^	Strain	A. Rainbow trout infection challenges				B. Ayu bath infection challenges			
		Trials	Infectious doses (CFU/mL)^b^		Total number of fish	Trials	Infectious doses (CFU/mL)^c^		Total number of fish
*P. altivelis*	AK-0527	I	1.0 × 10^6^	2.0 × 10^6^	60	IV	1.0 × 10^5^	1.4 × 10^5^	52
*O. mykiss*	SG950607	II	1.0 × 10^7^	4.0 × 10^6^	40	IV	7.0 × 10^4^	2.1 × 10^5^	70
*P. altivelis*	KU190628-77	I	2.3 × 10^6^	2.5 × 10^6^	60	I	8.0 × 10^5^	1.2 × 10^6^	60
						V	1.2 × 10^5 (*)^		59
						VI	4.7 × 10^4^	4.9 × 10^4^	60
*P. altivelis*	CS-1	I	4.2 × 10^6^	3.3 × 10^6^	60	III	4.5 × 10^5^	5.9 × 10^5^	59
*P. altivelis*	KU060626-4	I	1.6 × 10^6^	3.8 × 10^6^	60	V	5.8 × 10^4^	7.1 × 10^4 (*)^	64
						VI	3.2 × 10^5^	1.3 × 10^5^	59
*P. altivelis*	KU060626-59	I	2.0 × 10^6^	2.5 × 10^6^	60	V	1.6 × 10^5^	2.3 × 10^5^	65
						VI	3.1 × 10^5^	2.4 × 10^5^	59
*P. altivelis*	PH-0209	I	1.9 × 10^6^	2.2 × 10^6^	60	IV	3.4 × 10^5^	7.1 × 10^5^	53
*P. altivelis*	SG011227	I	1.7 × 10^6^	2.7 × 10^6^	60	IV	9.7 × 10^5^	6.4 × 10^5^	58
*P. altivelis*	FPC840	I	6.0 × 10^6^	5.7 × 10^6^	60	I	1.1 × 10^6^	1.1 × 10^6^	60
*P. altivelis*	KU 060626–56	I	1.5 × 10^6^	1.0 × 10^6^	60	I	7.0 × 10^5^	8.0 × 10^5^	57
						V	2.3 × 10^5^	2.5 × 10^4^	58
						VI	1.9 × 10^5 (*)^		60
*P. altivelis*	KU190628-79	I	3.3 × 10^6^	2.9 × 10^6^	60	I	9.0 × 10^5^	1.4 × 10^6^	60
						III	8.9 × 10^5^	7.2 × 10^5^	56
						IV	1.1 × 10^6^	8.6 × 10^5^	56
						V	1.6 × 10^3^	1.6 × 10^4^	59
						VI	6.2 × 10^4^	3.1 × 10^4^	60
*P. altivelis*	PH-0424	I	5.0 × 10^5^	6.0 × 10^5^	60	I	7.8 × 10^5^	8.6 × 10^5^	63
						II	1.9 × 10^6^	1.5 × 10^6^	64
						III	7.0 × 10^5^	7.7 × 10^5^	58
*P. altivelis*	KFCB-0566	II	2.8 × 10^6^	6 × 10^6^	40	V	3.8 × 10^5 (*)^		60
*C. carpio*	LFNW 16/90	I	2.3 × 10^6^	2.0 × 10^6^	60	II	2.5 × 10^6^	1.9 × 10^6^	60
						III	1.1 × 10^6^	9.7 × 10^5^	59
*O. mykiss*	FRGDSA 1882/11	I	1.2 × 10^6^		30				
		II	6.5 × 10^6^		20				
*O. mykiss*	BZ01	I	5.6 × 10^6^	7.0 × 10^6^	60	II	1.5 × 10^6^	1.8 × 10^6^	60
						III	1.2 × 10^6^	1.0 × 10^6^	52
*O. mykiss*	ENVN 740	I	6.1 × 10^6^	4.6 × 10^6^	60	II	8.9 × 10^5^	1.0 × 10^6^	61
						III	1.1 × 10^6^	9.7 × 10^5^	57
*S. salar*	DPIF 91/4043–17	I	1.0 × 10^5^	3.0 × 10^5^	60	II	1.2 × 10^6^	4.8 × 10^5^	60
						III	6.4 × 10^5^	5.2 × 10^5^	62

^a^Host species of origin of *F. psychrophilum* isolates: *P. altivelis* (*Plecoglossus altivelis*), *O. kisutch* (*Oncorhynchus kisutch*), *O. mykiss* (*Oncorhynchus mykiss*), *Z. platypus (Zacco platypus*), *H. nipponensis* (*Hypomesus nipponensis*), *T. hakonensis* (*Tribolodon hakonensis*), *C. carpio* (*Cyprinus carpio*), *O. masou* (*Oncorhynchus masou*), *S. leucomaenis (Salvelinus leucomaenis)*, *S. salar* (*Salmo salar*).

^b^For rainbow trout infection trials, bacterial concentration is indicated as CFU/mL of water for each duplicate tank at the end of immersion (T24).

^c^For ayu infection trials, bacterial concentration is indicated as CFU/mL of water for each duplicate tank at the beginning of immersion (T0); (*) when this data is not available, bacterial concentration in duplicated tanks is estimated based on the CFU counts of the bacterial culture used for infection.

### Ayu bath challenges

For ayu immersion challenges, 17 strains were tested through 6 trials using duplicated tanks (Table 1B). Ayu juvenile (body weight: 0.7~1.2 g) was purchased from Marinetech Co. Ltd. (Aichi, Japan). Usually, ayu are initially reared in seawater until 100~120 days post-hatching. *F. psychrophilum* is typically not viable in marine environments; therefore, initial screening of ayu for *F. psychrophilum* is unnecessary, as their marine rearing conditions naturally serve as a barrier against the bacterium’s presence. The salinity of the rearing water gradually decreases until it reaches freshwater levels for 2~3 days. In this experiment, we transported ayu from the hatchery to the laboratory with 1/3 strength seawater as it has been observed to improve the survival rates of ayu juveniles. To minimize stress, the same salinity level was maintained when introducing ayu into the aquaria. Thirty ayu were introduced into 10 L aquaria containing seawater at 1/3 strength and acclimated overnight. Subsequently, in order to renew and decrease the water salinity, a flow of dechlorinated and UV-treated tap water was initiated and maintained at a rate of 20 mL/min until the infection was conducted. Ten milliliters of fresh bacterial culture (OD600~0.3) were added to the aquaria containing 10L water and the bacterial concentration in water was determined by CFU counts on FLPA at the beginning of bacterial exposure (Table 1B). In ayu challenges, quantifying bacteria at 24 h post-exposure was not feasible due to environmental bacteria outcompeting *F. psychrophilum* in the water samples, resulting in technical difficulties in accurately determining the pathogen’s concentration through CFU counts on FLPA. As a negative control, 10 mL of sterile FLPB was added. Bacteria were maintained for 24 h. Then, 90% of water was replaced twice and the water flow was restarted and kept at a speed of 25 mL/min. NH_4_ levels were measured at the beginning of bacterial exposure and at the end, before refreshing the water. The temperature of the aquaria was 17.06 °C with a standard deviation of ± 0.95 °C throughout the experiments. Mortality was recorded daily for 14 days. The tissue homogenates of kidneys and spleen of dead fish were scraped off with a sterile loop and streaked onto FLPA. The inoculated FLPA was incubated at 18 °C for 4 days. Yellow colonies were analyzed using MALDI Biotyper and confirmed as *F. psychrophilum* using Bruker Realtime Classification software (Bruker Daltonics, Billerica, MA, USA).

### Statistical analyses

The Kaplan–Meier method was used to draw survival curves for each group of fish using combined data from all replicates (Table 1 and Additional file 1). Survival curves for fish infected with bacteria were compared with the survival curve for fish exposed to sterile FLPB (negative control) using the Mantel-Cox log-rank test with GraphPad Prism 8.1.2 (GraphPad Software, San Diego, CA, USA).

### Ethics statement

Rainbow trout experiments and sampling were performed at the INRAE-IERP fish facilities of Jouy-en-Josas (France) in accordance with the European Directive 2010/2063/UE. All animal work was approved by the Direction of the Veterinary Services of Versailles, France (building agreement number C78-720) and by the ethics committee of the INRAE Center in Jouy-en-Josas (COMETHEA n° 45), France (authorization numbers 2015100215242446). The ayu experiments were reviewed and approved by the animal care and use committee of Kindai University (Authorization KAAG2022-017). All methods are reported in accordance with ARRIVE guidelines.

## Results

### Molecular characterization of Japanese and outside Japanese isolates

In order to better characterize the genetic determinants underlying *F. psychrophilum* virulence and host association, we analyzed a collection of 37 strains retrieved in Japan from diverse host fish species (Table 2A). These strains were isolated between 1987 and 2019 from 10 different fish species. Most of the strains (32/37) were previously genotyped using MLST or WGS [9, 32]. We determined their Type O-Ag by mPCR serotyping (Table 2A). Strikingly, almost all strains (14 out of 15) retrieved from *P. altivelis* are Type-3 and the 3 strains retrieved from *O. kisutch* belong to Type-0. In contrast, strains retrieved from the other host species belong to various Type O-Ag as observed for the 5 strains retrieved from *O. mykiss* that are Type-0, Type-1, Type-2 and Type-3. The 4 strains retrieved from Cypriniformes belong to Type-0, Type-1 and Type-2 and none were Type-3. Indeed 15 out of 16 Type-3 strains were retrieved from Osmeriformes. We did not identify ayu-derived strains belonging to Type-1 nor Type-2, but 1 strain belongs to Type-0 (AK-0527), contrasting to the absolute Type-3 association previously noticed [20]. Among the 5 newly MLST typed isolates, 4 belong to ST52 and 1 to ST49. All isolates retrieved from ayu belong to the previously described ayu-associated CC-ST48-56 or CC-ST52 with one exception: strain AK-0527, the unique representative of ST53 in PubMLST database, was retrieved in Kyoto prefecture in 2005 from the lower jaw of a fish that did not show symptoms (Table 2A).

**Table 2:** **Serotyping, MLST profiles and virulence in rainbow trout and ayu for the *F. psychrophilum* strains used in the study**

Strains^(1)^	Species origin ^(2)^	Host order origin	Year	Region	Serotyping mPCR	MLST (STs, CC and allele type profiles)									Virulence in rainbow trout^(6)^	Virulence in ayu^(6)^
					Type^(3)^	ST^(4)^	CC^(5)^	*atpA*	*dnaK*	*fumC*	*gyrB*	*murG*	*trpB*	*tuf*		
A. Strains isolated in Japan																
AK-0527*	*P. altivelis*	Osmeriformes	2005	Kyoto	0	53^a^	Singleton	28	19	2	19	13	19	29	No	No
x-2	*O. kisutch*	Salmoniformes	2005	Iwate	0	30^a^	Singleton	17	7	5	18	14	11	18		
FPM960724	*O. kisutch*	Salmoniformes	1996	Miyagi	0	30^a^	Singleton	17	7	5	18	14	11	18		
FPM960726	*O. kisutch*	Salmoniformes	1996	Miyagi	0	9^a^	ST9	4	6	5	7	6	4	5		
y-3	*O. mykiss*	Salmoniformes	2005	Iwate	0	42^a^	Singleton	23	4	5	25	18	4	23		
SG010808	*O. mykiss*	Salmoniformes	2001	Shiga	0	47^a^	Singleton	26	15	12	29	17	2	27		
CH-0411	*Carassius* spp*.*	Cypriniformes	2004	Hiroshima	0	51^a^	ST48-56	8	8	6	24	8	5	1		
ZH-0001	*Z. platypus*	Cypriniformes	2000	Hiroshima	0	51^a^	ST48-56	8	8	6	24	8	5	1		
0312	*O. mykiss*	Salmoniformes	2003	Yamanashi	1	50^a^	Singleton	21	4	5	28	21	7	28		
SG980216	*H. nipponensis*	Osmeriformes	1998	Shiga	1	44^a^	Singleton	25	10	7	27	20	18	25		
SG040302	*H. nipponensis*	Osmeriformes	2004	Shiga	1	57^a^	Singleton	28	19	2	31	23	20	30		
GM2127	*T. hakonensis*	Cypriniformes	1999	Gunma	1	46^a^	Singleton	4	8	11	28	1	4	26		
CH-9401	*C. carpio*	Cypriniformes	1994	Hiroshima	1	51^a^	ST48-56	8	8	6	24	8	5	1		
SG950607*	*O. mykiss*	Salmoniformes	1995	Shiga	2	10^a^	ST10	2	2	2	8	2	2	2	High	No
y-2	*O. mykiss*	Salmoniformes	2005	Iwate	2	10^a^	ST10	2	2	2	8	2	2	2		
OH-0203	*O. mykiss*	Salmoniformes	2002	Hiroshima	2	10^a^	ST10	2	2	2	8	2	2	2		
OH-0224	*O. masou*	Salmoniformes	2003	Hiroshima	2	54^a^	ST54	23	20	2	30	22	2	22		
OH-0519	*O. masou*	Salmoniformes	2005	Hiroshima	2	55^a^	ST54	23	20	2	30	23	2	22		
SG020617	*S. leucomaenis*	Salmoniformes	2002	Shiga	2	41^a^	Singleton	22	17	5	4	10	16	12		
PH-9348	*Z. platypus*	Cypriniformes	1993	Hiroshima	2	58^a^	Singleton	29	10	7	32	24	21	31		
KU190628-77*	*P. altivelis*	Osmeriformes	2019	Shiga	3	49	ST48-56	27	2	6	24	8	5	1	No	Moderate
CS-1*	*P. altivelis*	Osmeriformes	1995	Gifu	3	45^a^	ST48-56	8	19	5	24	8	19	1	No	No
KU060626-4*	*P. altivelis*	Osmeriformes	2006	Shiga	3 ^a^	49^c^	ST48-56	27	2	6	24	8	5	1	No	No
KU060626-59*	*P. altivelis*	Osmeriformes	2006	Shiga	3 ^a^	48^b^	ST48-56	8	19	6	24	8	19	1	No	Moderate
PH-0209*	*P. altivelis*	Osmeriformes	2002	Hiroshima	3	48^a^	ST48-56	8	19	6	24	8	19	1	Moderate	Moderate
SG011227*	*P. altivelis*	Osmeriformes	2001	Shiga	3	56^a^	ST48-56	8	2	6	24	8	5	1	No	Low
PH9351	*P. altivelis*	Osmeriformes	1993	Hiroshima	3	45^a^	ST48-56	8	19	5	24	8	19	1		
PH-0003	*P. altivelis*	Osmeriformes	2000	Hiroshima	3	45^a^	ST48-56	8	19	5	24	8	19	1		
FPC 840*	*P. altivelis*	Osmeriformes	1987	Tokushima	3 ^a^	5^c^	ST52	4	4	4	4	2	5	4	No	Moderate
KU060626-56*	*P. altivelis*	Osmeriformes	2006	Shiga	3	52	ST52	4	4	4	24	2	5	4	No	Moderate
KU190628-79*	*P. altivelis*	Osmeriformes	2019	Shiga	3	52	ST52	4	4	4	24	2	5	4	Low	High
PH-0424*	*P. altivelis*	Osmeriformes	2004	Hiroshima	3	52	ST52	4	4	4	24	2	5	4	No	Moderate
KFCB-0566*	*P. altivelis*	Osmeriformes	2018	Kochi	3	52	ST52	4	4	4	24	2	5	4	Moderate	High
96–4	*P. altivelis*	Osmeriformes	1996	Gifu	3	52^a^	ST52	4	4	4	24	2	5	4		
CS-3	*O. mykiss*	Salmoniformes	1995	Gifu	3	39^a^	Singleton	2	4	10	23	6	14	22		
SG010619	*H. nipponensis*	Osmeriformes	2001	Shiga	3	52^a^	ST52	4	4	4	24	2	5	4		
SG030207	*O. masou*	Salmoniformes	2003	Shiga	4	40^a^	Singleton	11	10	3	24	2	15	12		
B. Strains isolated outside Japan																
LFNW 16/90*	*C. carpio*	Cypriniformes	1990	Germany	0^a^	14^c^	ST48-56	8	8	6	10	8	5	1	Low	No
FRGDSA 1882/11*	*O. mykiss*	Salmoniformes	2011	France	2^a^	108^b^	ST90	1	1	2	1	1	1	41	High	n.d
BZ 01*	*O. mykiss*	Salmoniformes	1998	Israel	3^a^	16^c^	ST10	2	2	2	8	2	2	3	High	No
ENVN 740*	*O. mykiss*	Salmoniformes	2009	France	3^a^	2	ST10	2	2	2	2	2	2	2	High	No
DPIF 91/4043–17*	*S. salar*	Salmoniformes	1991	Tasmania	3^a^	7^c^	Singleton	5	4	3	5	4	4	5	Low	Low

^(1)^The 18 strains used in the experimental infection trials are indicated with an asterisk*.

^(2)^*P. altivelis* (*Plecoglossus altivelis*), *O. kisutch* (*Oncorhynchus kisutch*), *O. mykiss* (*Oncorhynchus mykiss*), *Z. platypus (Zacco platypus*), *H. nipponensis* (*Hypomesus nipponensis*), *T. hakonensis* (*Tribolodon hakonensis*), *C. carpio* (*Cyprinus carpio*), *O. masou* (*Oncorhynchus masou*), *S. leucomaenis (Salvelinus leucomaenis)*, *S. salar* (*Salmo salar*).

^(3)^A, serotype mPCR data of 8 strains are from [20].

^(4)^MLST data are from: ^a^, [9]; ^b^, [32]; ^c^, [8].

^(5)^Isolates sharing at least 5 ATs were assigned to the same clonal complex (linked by single and double locus variants).

^(6)^Strains belong to 4 categories of virulence, defined as follows: “No”, strains not pathogenic considering the absence of statistical difference between Kaplan–Meier survival curves of infected and mock-exposed groups (Mantel-Cox log-rank test *p*-value ≥ 0.05); for the 3 other categories of virulence, survival curves are statistically different (Mantel-Cox log-rank test *p*-value < 0.05) and the final percentage of survival is < 50% for “High”, between 50 and 85% for “Moderate” and > 85% for “Low” virulence categories. Detailed results of survival analyses are available in the Additional files 1 and 2.

### Comparing virulence of *F. psychrophilum* strains in rainbow trout and ayu

**Figure 1:**
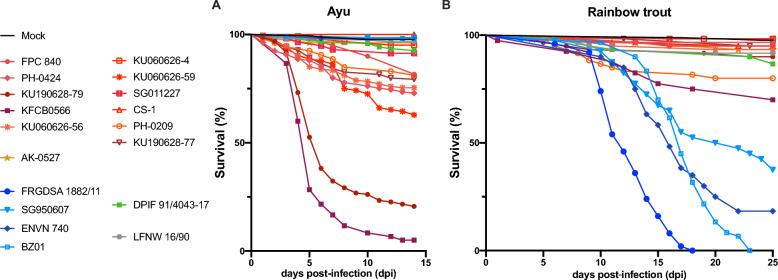
**Survival kinetics of ayu and rainbow trout following infection by bath with diverse *F. psychrophilum* strains.** Kaplan–Meier survival curves of ayu (**A**) and rainbow trout (**B**) are plotted for each strain using the combined data from all trials described in Table 1. Individual representations of the survival curves for each strain and the mock control group, along with corresponding *p*-values of the log-rank Mantel-Cox test, are available in the Additional files 1 and 2. Colors indicate fish host origin: ayu (brown-to-red); rainbow trout (blue); Atlantic almon (green); carp (grey).

In order to explore relationships between serotype, ST and host association, we compared the virulence in 2 hosts, ayu and rainbow trout, for a selection of *F. psychrophilum* strains of diverse origins and genotypes. A set of 13 strains originated from Japan (Table 2A): 1 strain (SG950607) was retrieved from rainbow trout and belongs to Type-1 and CC-ST10; 12 were retrieved from ayu, among which all possess Type-3 O-Ag except strain AK-0527 that is Type-0, 6 of them belong to CC-ST48-56 and 5 to CC-ST52. In addition, 4 strains isolated outside Japan were selected to help addressing the respective roles of O-Ag and ST in virulence (Table 2B): 2 rainbow trout-derived strains possess a Type-3 O-Ag and belong to CC-ST10; 1 strain (DPIF 91/4043–17) retrieved from Atlantic salmon is Type-3 and ST7; and 1 strain (LFNW 16/90) isolated from carp in Germany—categorized Type-0 and ST14 (CC-ST48-56)—was selected due to its phylogenetic proximity to strains associated to BCWD outbreaks in Japan. For rainbow trout infection challenges, strain FRGDSA 1882/11 isolated in France from rainbow trout and possessing Type-2 O-Ag, was used as a highly virulent control strain. Comparison of survival curves of fish infected by bath with this selection of strains showed high variation in virulence in both ayu and rainbow trout (Figure 1). Pathogenicity was assessed by analyzing differences in survival curves between groups infected by each strain and the mock-exposed control group (individual representations of survival curves for each strain and *p*-values are available in the Additional files 1 and 2). Strains that produced significant mortality (*p*-value < 0.05) were then categorized in 3 groups based on the final percentage of survival, as high (<50%), moderate (50–85%) and low (>85%) virulence (Table 2).

In ayu, 7 strains did not produce significant mortality (Figure 1A, Table 2, Additional files 1 and 2). They were retrieved from rainbow trout (ENVN740, BZ01 and SG950607), Atlantic salmon (LFNW16/90) and ayu (AK-0527, KU060626-4, CS-1). Strikingly, all strains with high (KU190628-79 and KFCB-0566) or moderate (KU190628-77, KU060626-59, FPC840, PH-0209, PH-0424, KU060626-56) virulence were isolated from ayu, belonged to CC-ST52 or CC-ST48-56, and displayed a Type-3 O-Ag. All the strains (6) not belonging to those 2 CC, whatever the fish host origin (including ayu), displayed low or no virulence in ayu, even if possessing Type-3 O-Ag. The unique strain not retrieved from ayu (DPIF 91/4043–17) that produced statistically significant—though low—mortality (6.6%, *p*-value = 0.0097; Additional files 1 and 2) in ayu displayed a Type-3 O-Ag. These data suggest that virulence in ayu requires strains at least possessing Type-3 O-Ag and belonging to CC-ST52 or CC-ST48-56. Nevertheless, these criteria are not always sufficient in themselves as 2 strains (KU060626-4 and CS-1) belonging to CC-ST48-56 and possessing Type-3 O-Ag were not pathogenic for ayu. In addition, our results show that the most highly virulent strains were recently isolated (2018 and 2019) and belonged to CC-ST52. Of importance, Type-3 O-Ag seems required but not sufficient on its own for virulence in ayu as other strains belonging to this CC have moderate (KU060626-56 and PH-0424) or low (FPC 840) virulence, though all were isolated many years ago.

In the rainbow trout challenge, 9 out of 18 strains produced significant mortality (Figure 1B, Table 2 and Additional files 1 and 2). Strikingly, the 4 highly virulent strains were all retrieved from rainbow trout and belonged to CC-ST10 (ENVN740, BZ01 and SG950607) or CC-ST90 (FRGDSA 1882/11). Interestingly, 2 of these strains possessed a Type-3 O-Ag. Among the 2 strains with moderate virulence, one belonged to CC-ST52 (KFCB-0566) and the other to CC-ST48-56 (PH-0209), both displayed a Type-3 O-Ag and were isolated from ayu. Strains with low virulence in rainbow trout were derived from ayu (KU190628-79), Atlantic salmon (DPIF91/4043–17) and carp (LFNW16/90). In conclusion, all strains belonging to rainbow-trout associated CC displayed high virulence in rainbow trout independently of their O-Ag whereas most of ayu-derived strains (9/12) were not pathogenic for rainbow trout in the bath infection model.

## Discussion

*F. psychrophilum* has currently a worldwide distribution and salmonids, especially rainbow trout and Atlantic salmon, are particularly affected species. However, *F. psychrophilum* has been frequently associated to diseased ayu (an Osmeriformes fish) in farms and rivers [4]. In addition, *F. psychrophilum* has been occasionally isolated from other non-salmonid freshwater fish [1]. Therefore, the range of natural host species remains unclear. Isolates sampling is biased toward fish farms and only few publications mentioned *F. psychrophilum* in natural fish populations [14, 33]. Intrinsic host resistance/susceptibility should also be considered and the use of rainbow trout isogenic lines or full-sib families has revealed considerable differences inside a single host species [34, 35].

Different typing methods such as random amplification of polymorphic DNA, PCR-restriction fragment length polymorphism, ribotyping, pulsed-field gel electrophoresis, conventional serotyping, and plasmid profiling have been used to characterize the population structure of *F. psychrophilum.* More recently, MLST, mPCR serotyping and complete genome sequencing were proven to be effective and reliable strategies for meaningful strain comparisons and phylogenomic analyses enabled the identification of strong relationships between certain bacterial genotypes and their hosts [8, 15, 20, 32]. However, these associations are not absolute. Therefore, it remains to be elucidated the respective contributions of pathogen characteristics (i.e., genetic determinants of virulence and host specificity) and fish susceptibility on the success and severity of the infection.

Molecular determinants responsible for *F. psychrophilum* host specificity remain unclear. Nakayama et al. identified association between the presence of the collagenase encoding gene in *F. psychrophilum* isolates and BCWD in ayu, but the direct role of this gene in virulence was not evaluated using fish infection experiments [36]. On the other hand, Castillo et al. stressed no clear association between genomic repertoire, phylogeny and virulence in rainbow trout when focusing on a selection of rainbow trout-derived strains [37]. O-antigen can play an important role in pathogenesis, contributing to several steps of the infection process, such as adherence required for host colonization or resistance to host defense mechanisms [38]. Our former study revealed a striking association between mPCR-serotype and host fish species suggesting that the nature of the O-Ag provides a selective advantage according to the infected host species [20]. Indeed, all *F. psychrophilum* strains previously isolated from ayu possess Type-3 O-Ag as opposed to 6% isolates from rainbow trout (8 from France and one from Israel out of 151). However, the virulence of these strains was evaluated neither in ayu nor in rainbow trout.

Previous virulence studies addressing the question of *F. psychrophilum* host specificity focused on a few strains tested in several fish species [24, 26, 39–42] or on a single host challenged with isolates retrieved from various fish species [23, 25, 42, 43]. However, the limited number of strains tested in each study and the lack of data regarding their genotype make comparisons difficult. Challenges performed in Atlantic salmon with rainbow trout-derived isolates resulted either in high or no mortality [23, 25, 26, 43]. Bruce et al. [26] evaluated simultaneously the virulence in Atlantic salmon and brook trout (*Salvelinus fontinalis*) of 4 different, MLST-characterized strains: CSF 259–93 (ST10) isolated from and known to be virulent in rainbow trout; US063 (ST278) isolated from lake trout (*Salvelinus namaycush*); 03–179 (ST294; CC-ST10) isolated from steelhead trout (*Oncorhynchus mykiss*) and US149 (ST70; CC-ST124) isolated from Atlantic salmon. Some conclusions were drawn such as that Atlantic salmon may be resistant to some STs or *F. psychrophilum* strains regardless of host origin and that *F. psychrophilum* isolates originating from rainbow trout have the potential to cause disease in brook trout. Knupp and Loch recently provided in vivo experimental evidence of host-specificity among the *F. psychrophilum* genotypes [24]. Bath challenges performed in 3 host species (coho salmon, Atlantic salmon and rainbow trout) with 3 strains originally retrieved from those 3 hosts and belonging to different genotypes (namely, CC-ST9 for strain US19-COS, CC-ST232 for US62-ATS and CC-ST10 for US87-RBT) revealed important variations in disease development and subsequent mortality. Strikingly, the rainbow trout-derived isolate was only pathogenic for rainbow trout. Conversely, US19-COS and US62-ATS induced higher mortality in both coho and Atlantic salmon, and to a lesser extent in rainbow trout, suggesting the influence of specific antigenic or virulence factors [24]. These results are in accordance with the findings from our comparative virulence study in ayu and rainbow trout hosts. In a preliminary study performed on 2 strains also characterized herein, we observed that the ayu-derived strain PH-0424 was able to grow in vitro in ayu serum whereas the rainbow trout-derived strain SG950607 did not, suggesting that host specificity of *F. psychrophilum* strains may be partly supported by their different ability to resist to the complement [40].

In this study, we explored relationships between serotype, ST and virulence by analyzing a collection of *F. psychrophilum* strains retrieved from diverse fish species in Japan using mPCR serotyping and MLST (Table 2). The results are in good accordance with our previous observations regarding association between host fish species and serotypes [20]. We indeed observed that most strains retrieved from ayu belong to Type-3 O-Ag whereas strains retrieved from rainbow trout belong to Type-0, Type-1 and Type-2 but also to Type-3 O-Ag. However, we identified for the first time 1 strain retrieved from ayu and not possessing Type-3 O-Ag. The Japanese strains also displayed an important heterogeneity based on MLST and the presence of different *F. psychrophilum* lineages in Japan, at least 2 of which (i.e., CC-ST52 and CC-ST48-56) infecting ayu, was confirmed [9].

On the basis of fish host origin, MLST genotype and O-Ag type, we selected 17 strains belonging to different genetic groups and compared their virulence in rainbow trout and ayu using experimental infection by bath, a model that mimics the natural route of infection. In ayu, only CC-ST52 and O-Ag Type-3 strains were highly virulent; only CC-ST52 or CC-ST48-56 and O-Ag Type-3 strains were moderately virulent, while strains not possessing this combination were poorly or not virulent at all. This suggests a rather restricted genetic landscape for *F. psychrophilum* strains able to infect (or at least to be virulent to) ayu. As this limited combination was yet only observed in Japanese strains, this observation goes against the hypothesis of a recent introduction of *F. psychrophilum* in Japan*,* at least for the ayu-specific lineages, as previously suggested using a MLST-based epidemiology survey [9]. However, this association was not absolute since CC-ST48-56 and O-Ag Type-3 strains (KU060626-4 and CS-1) displayed no virulence in ayu. Although putative virulence attenuation during in vitro manipulation of those old isolates cannot be excluded, this result may be the consequence of other genes presence/absence of polymorphisms not captured using MLST and mPCR typing schemes. Indeed, the *F. psychrophilum* O-Ag encoding loci are highly diverse and mPCR serotyping only capture the major molecular determinants (i.e., the *wzy* gene encoding the polymerase) but other variations were reported such as differences in genes predicted to direct the synthesis of different R-groups [20, 44]. The current challenge experiments did not use CC-ST52 strains belonging to Type-1 or -2, which have not been found so far. Evaluating the virulence of such strain would be of interest in the future.

Only the 4 strains retrieved from rainbow trout were highly virulent in rainbow trout. Surprisingly, ayu-derived strains belonging to CC-ST52 (KFCB-0566) and CC-ST48-56 (PH0209) were moderately virulent in rainbow trout. This could be attributed to the fact that the rainbow trout isogenic line used for the bath challenge is highly susceptible to BCWD. Previous studies using intramuscular injection challenge in rainbow trout reported that strains belonging to Type-1 and Type-2 are highly virulent [21]. The current study showed that Type-3 strains isolated from rainbow trout are also highly virulent in rainbow trout following bath infection. Therefore, the virulence of *F. psychrophilum* in rainbow trout seems rather independent of the O-Ag Type (at least for Type-1, -2 and -3 strains) but instead more likely correlated to CC/ST as highly virulent strains belonged to the well-known CC-ST10.

Infection is a complex process encompassing a pathogen (with genetic variability), different host species (each with genetic differences) and the environment. In this study, we analyzed *F. psychrophilum* variability using MLST and mPCR serotyping and performed experimental challenges using 2 BCWD susceptible hosts for a selection of strains. Our results revealed that isolates of *F. psychrophilum* can display significant variation in virulence according to the host. Striking association trends were observed that will require future genome mining to identify subtle traits associated with virulence and host range. This study could also pave the way for a better understanding of co-infection of the same host by strains with unconnected genotype and serotype. Indeed, *F. psychrophilum* co-infections might be frequent [7, 9, 45] and grabbing the relative contribution of each bacterium involved in this process will require extensive knowledge using single strain assessment. Finally, deciphering the interactions between host species susceptibility to BCWD and *F. psychrophilum* genotypes is a prerequisite for the rational development of control strategies, such as vaccines or selective breeding for resistant hosts.

### Supplementary Information

**Additional file 1**. **Comparing virulence of *F. psychrophilum* strains in rainbow trout and ayu.** Kaplan-Meier survival curves of ayu (left panel) and rainbow trout (right panel) drawn using combined data from all trials listed in Table 1. Symbols and color codes are kept identical as Figure 1. Error bars represent 95% confidence intervals and statistical significance (Mantel-Cox logrank test) is indicated by *p*-values (ns, not significative).

**Additional file 2**. **Survival rate of rainbow trout and ayu after bath infection for each strain compared to the non-infected control (mock).**
