# Supplementary figures and images for: Host specificity and virulence of Flavobacterium psychrophilum: a comparative study in ayu (Plecoglossus altivelis) and rainbow trout (Oncorhynchus mykiss) hosts
Source: Vet Res. 2024 Jun 12;55:75. doi: 10.1186/s13567-024-01326-6 (PMC11167770; doi:10.1186/s13567-024-01326-6)

**
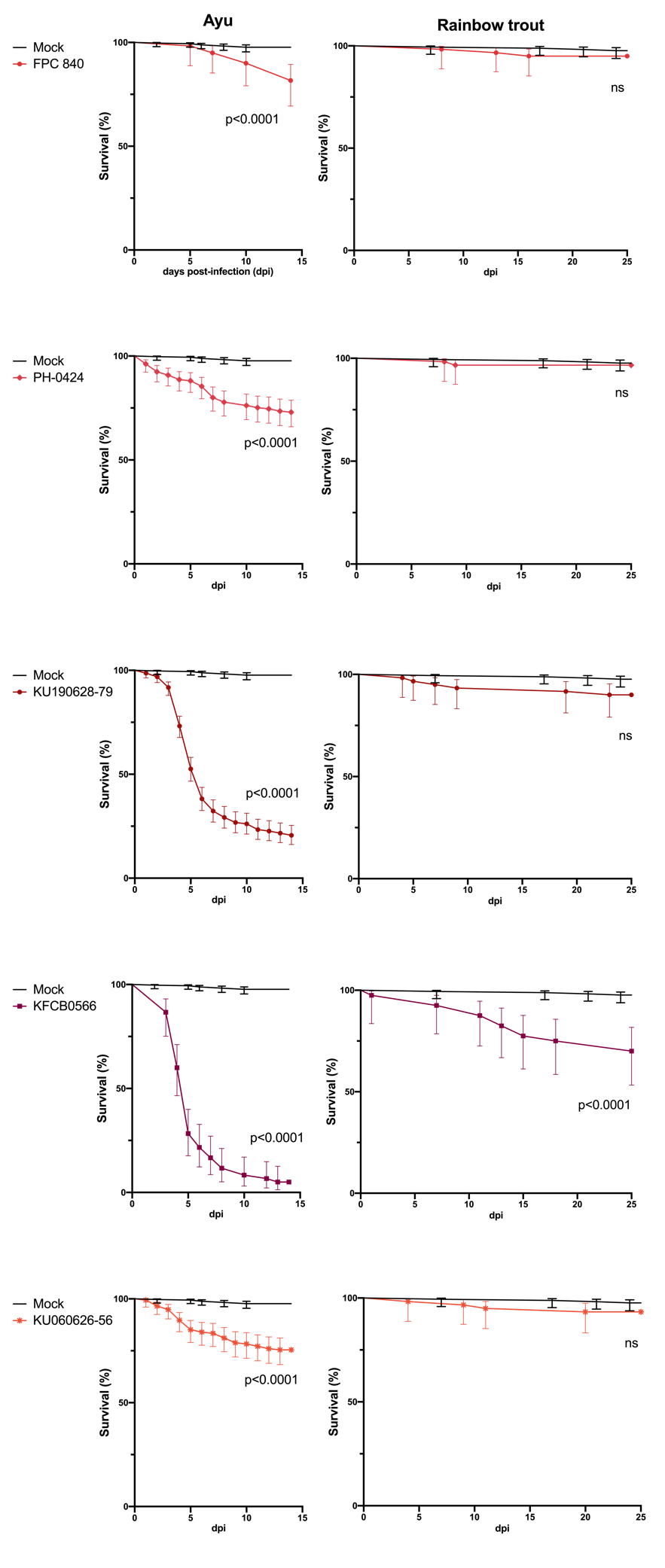
**

*
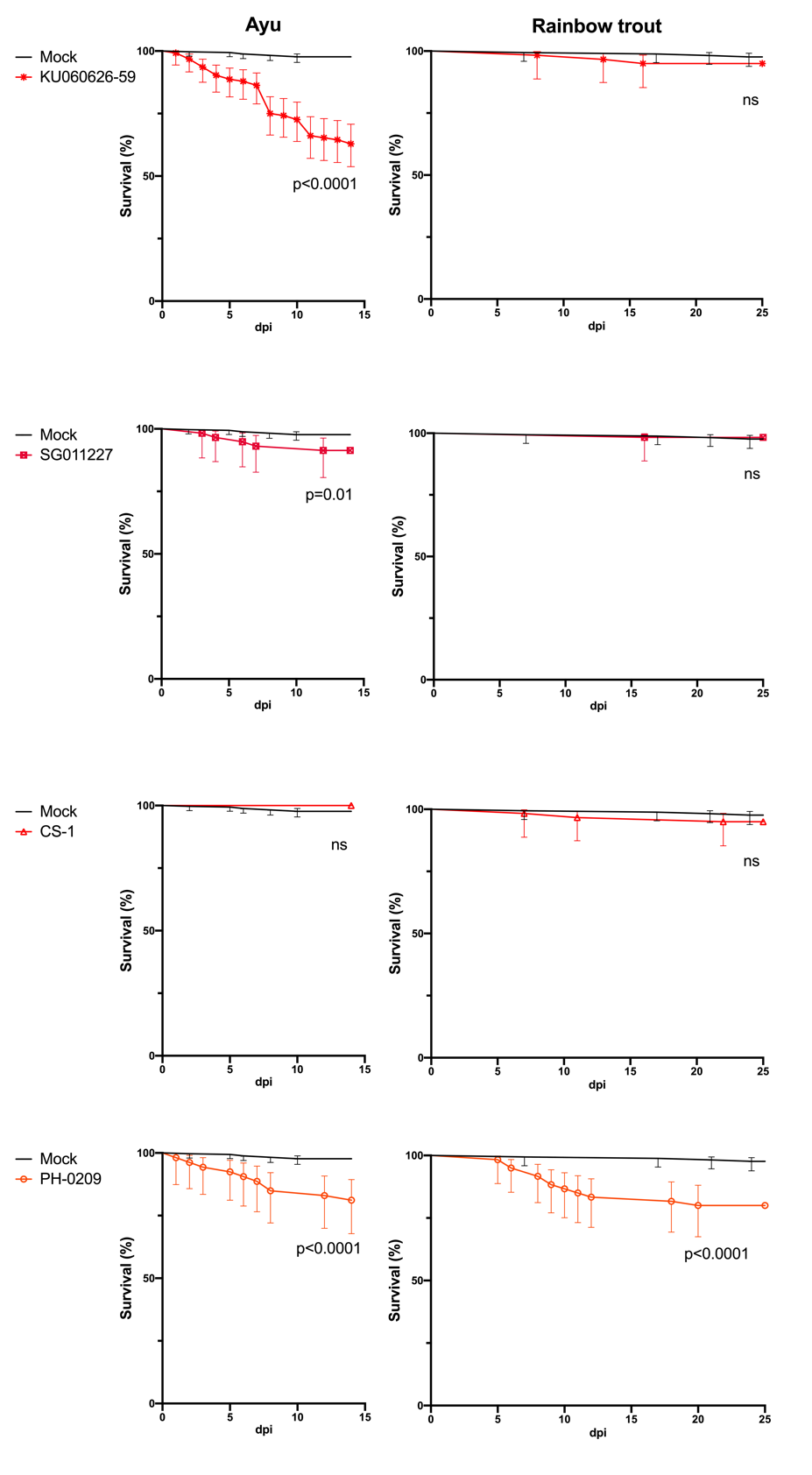
*


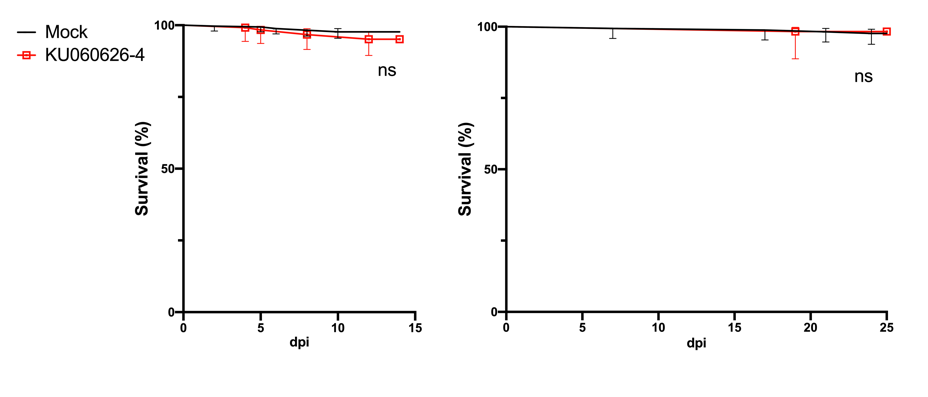


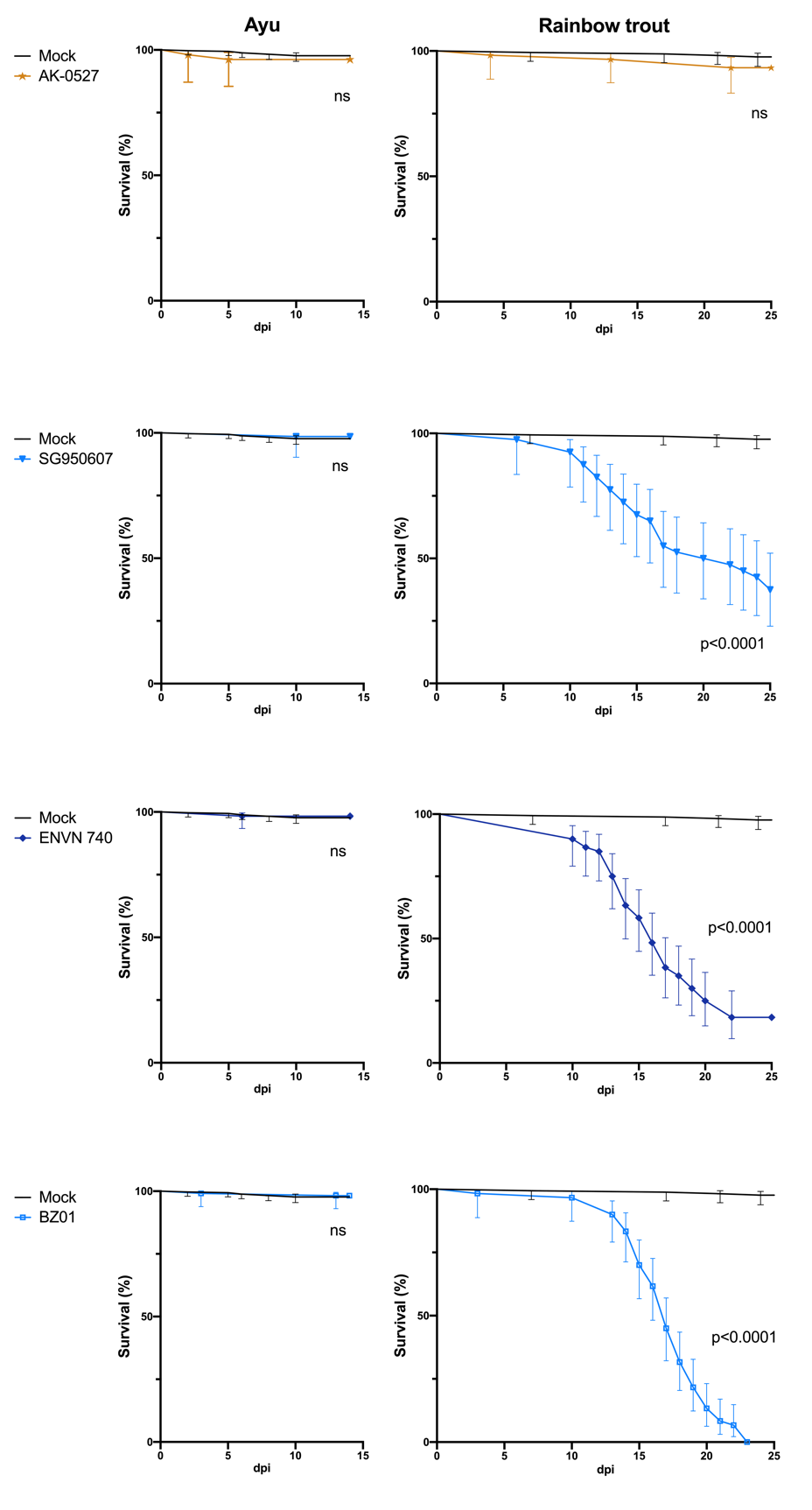


*
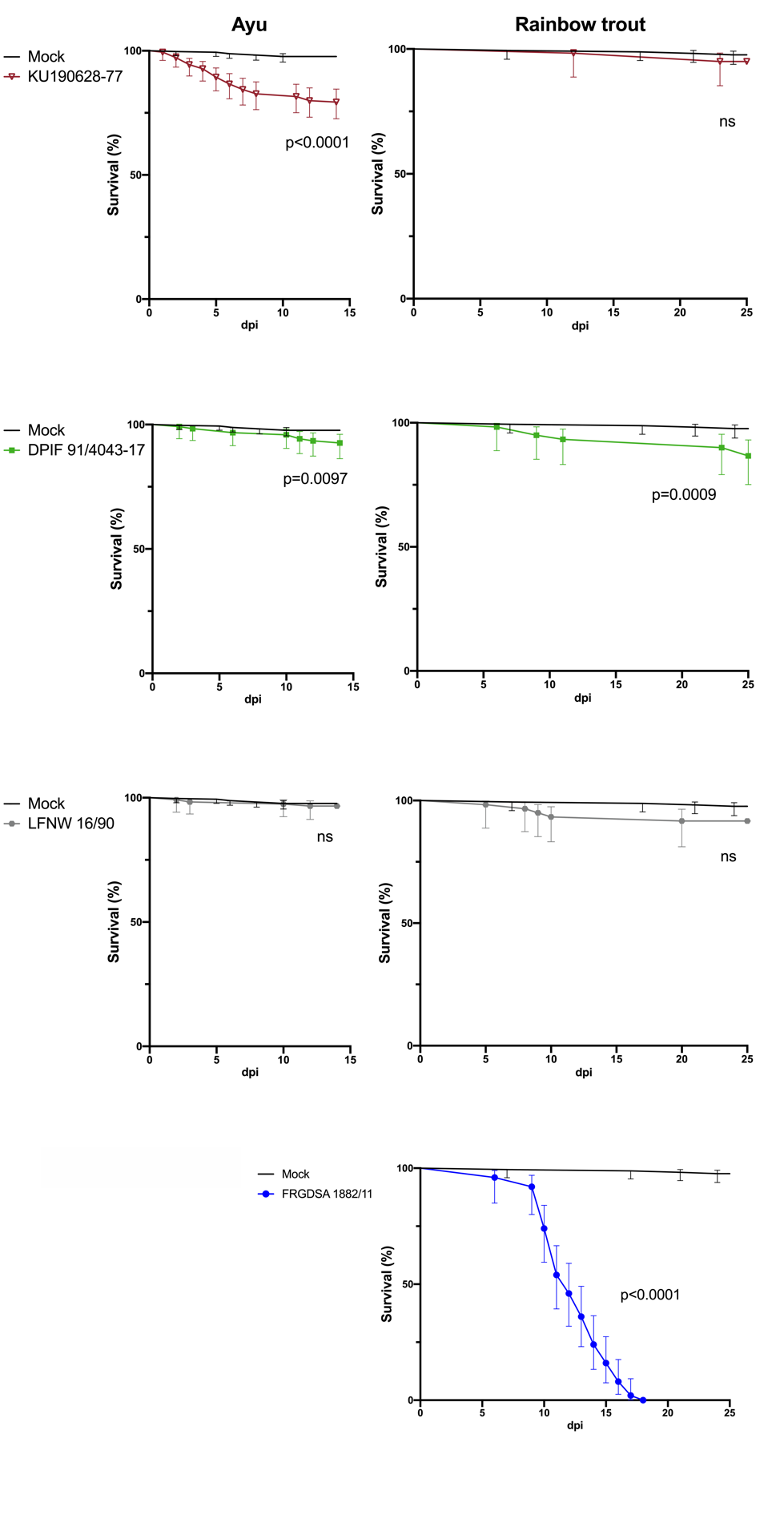
*

Supplement: Supplementary file 1 — Additional file 1. Comparing virulence of F. psychrophilum strains in rainbow trout and ayu. Kaplan-Meier survival curves of ayu (left panel) and rainbow trout (right panel) drawn using combined data from all trials listed in Table 1. Symbols and color codes are kept identical as Figure 1. Error bars represent 95% confidence intervals and statistical significance (Mantel-Cox logrank test) is indicated by p-values (ns, not significative). [file 13567_2024_1326_MOESM1_ESM.docx]
