# Supplementary material for: Host specificity and virulence of Flavobacterium psychrophilum: a comparative study in ayu (Plecoglossus altivelis) and rainbow trout (Oncorhynchus mykiss) hosts
Source: Vet Res. 2024 Jun 12;55:75. doi: 10.1186/s13567-024-01326-6 (PMC11167770; doi:10.1186/s13567-024-01326-6)
Supplement: Supplementary file 2 — Additional file 2. Survival rate of rainbow trout and ayu after bath infection for each strain compared to the non-infected control (mock). [file 13567_2024_1326_MOESM2_ESM.docx]

**Additional file 2. Survival rate of rainbow trout and ayu after bath infection for each strain compared to the non-infected control (mock).**
